# SARS-CoV-2 variants and effectiveness of vaccines: a review of current evidence

**DOI:** 10.1017/S0950268821002430

**Published:** 2021-11-04

**Authors:** Elizabeth-Barbara Tatsi, Filippos Filippatos, Athanasios Michos

**Affiliations:** First Department of Pediatrics, Infectious Diseases and Chemotherapy Research Laboratory, Medical School, National and Kapodistrian University of Athens, ‘Aghia Sophia’ Children's Hospital, 11527 Athens, Greece

**Keywords:** Severe acute respiratory syndrome coronavirus 2, spike mutations, vaccines, variants of concern

## Abstract

The SARS-CoV-2 virus is rapidly evolving via mutagenesis, lengthening the pandemic, and threatening the public health. Until August 2021, 12 variants of SARS-CoV-2 named as variants of concern (VOC; Alpha to Delta) or variants of interest (VOI; Epsilon to Mu), with significant impact on transmissibility, morbidity, possible reinfection and mortality, have been identified. The VOC Delta (B.1.617.2) of Indian origin is now the dominant and the most contagious variant worldwide as it provokes a strong binding to the human ACE2 receptor, increases transmissibility and manifests considerable immune escape strategies after natural infection or vaccination. Although the development and administration of SARS-CoV-2 vaccines, based on different technologies (mRNA, adenovirus carrier, recombinant protein, etc.), are very promising for the control of the pandemic, their effectiveness and neutralizing activity against VOCs varies significantly. In this review, we describe the most significant circulating variants of SARS-CoV-2, and the known effectiveness of currently available vaccines against them.

## Introduction

Coronaviruses (CoVs) are enveloped positive-sense single-stranded RNA viruses of *Coronaviridae* family, which infect both animals and humans [1, 2]. Six human CoVs, HCoV-229E, HCoV-NL63, HCoV-HKU1, HCoV-OC43, Middle East respiratory syndrome coronavirus (MERS-CoV) and severe acute respiratory syndrome coronavirus (SARS-CoV), were known to cause upper and lower respiratory tract infections. However, severe outbreaks had been exacerbated by SARS-CoV and MERS-CoV in 2003 and 2012, respectively [3].

In 2019, a novel CoV was identified as the causative agent of a significant number of pneumonia cases in Wuhan, China. In February 2020, this CoV strain was named SARS-CoV-2 and the disease as Coronavirus Disease 2019 (COVID-19) by the World Health Organization (WHO) [4]. SARS-CoV-2 rapidly spread worldwide causing a pandemic.

To date, SARS-CoV-2 was closely related to two bat CoVs, the bat-SL-CoVZC45 (87.99%) and the bat-SL-CoVZXC21 (87.23%), but it was more distant from SARS-CoV (79%) and MERS-CoV (50%) [5]. Even though it is likely bats are the primary source for SARS-CoV-2 transmission, it remains unknown whether it is directly transmitted from bats to humans or through an intermediate host [6]. Recently, the most relative CoV was found in two *Rhinolophus shameli* bats in Cambodia with 92.6% nucleotide identity [7].

SARS-CoV-2 genome encodes four structural proteins: spike (S), envelope (E), membrane (M) and nucleocapsid (N); and 16 non-structural proteins (NSPs) [8]. The viral glycoprotein S is involved in the virus entrance to human cells by binding to the same receptor as SARS-CoV, the angiotensin-converting enzyme 2 (ACE2) [5, 9].

Specifically, S protein is comprised of the S1 subunit, which contains the receptor-binding domain (RBD), and the S2 subunit that mediates the virus fusion with the host cell membrane after its trimming mainly by the trans-membrane serine protease 2 (TMPRSS2) [10, 11]. RBD region is the primary target of neutralizing antibodies (NAbs) and cytotoxic lymphocytes, even though there are other regions of S protein that stimulate neutralizing activity (NAc) as well [12].

Although the NSP14 of CoVs acts as 3′-5′ exoribonuclease resulting in decreased variant gathering compared to other RNA viruses, the rapid spread of SARS-CoV-2 worldwide enhances the mutagenesis of viral genome [13, 14]. The majority of the variants have no impact on viral function. However, there are certain variants, especially in the spike protein of SARS-CoV-2, that have gained widespread attention, mainly due to their impact on ACE2 binding, TMPRSS2 cleavage or escape from immunity which alter the transmissibility, antigenicity, morbidity, clinical symptoms and implications or decrease the response to potential treatment [15].

These variants were called variants of concern (VOCs) and variants of interest (VOIs). The name and monitoring of these variants was performed by the World Health Organization (WHO; www.who.int/), genomic databases: Global Initiative on Sharing All Influenza Data (GISAID; www.gisaid.org/) [16] and Nextstrain (nextstrain.org/) [17] as well as the epidemiological tool Phylogenetic Assignment of Named Global Outbreak Lineages (PANGOLIN; cov-lineages.org/) [18]. All these databases freely share genomic data and serve the direct surveillance of all these new and significant variants.

Until the beginning of August 2021, four VOCs (Alpha to Delta) and six VOIs (Epsilon to Lambda) were detected. On 30 August 2021, a novel variant of SARS-CoV-2 was designated as Mu variant and it was also classified as VOI by WHO [19]. In September 2021, the variant classification system was changed and according to WHO, the variants were distinguished in VOCs (Alpha to Delta), VOIs (Lambda and Mu) and Variants Under Monitoring (VUM) [19]. The last group includes all the other variants, previously reported as VOIs, excluding the Zeta and Theta [19].

COVID-19 vaccines are being developed based on several different platforms, either with traditional approaches, such as live attenuated viruses, or with novel techniques, such as recombinant proteins and mRNA. The development and administration of SARS-CoV-2 vaccines are very promising for the control of the pandemic, nevertheless their effectiveness and NAc against VOCs and VOIs vary significantly [20].

For the purposes of this review, we describe the most significant VOCs and VOIs of SARS-CoV-2 based on the WHO classification of August 2021, including the novel Mu variant, as well as how effective is the currently available vaccines against these circulating variants.

## Variants of SARS-CoV-2

### Alpha VOC

In December of 2020, the first VOC of SARS-CoV-2 emerged in the UK, England. This new SARS-CoV-2 strain belongs to B.1.1.7 lineage, it is derived from the 20I (V1) clade, and it was named as Alpha VOC by WHO.

This VOC contains seven missense mutations all over the Spike gene and three deletions (p.H69del, p.V70del and p.Y144del) within the N-terminal domain (NTD) of the S1 subunit of the Spike (Table 1). The complete deletion of amino acids at positions 69–70 of the spike protein is the result of a six-nucleotide deletion, which probably affects the viral recognition by NAbs [21]. The deletion at position 144 is the result of a four-nucleotide deletion which is also associated with antibody escape [22]. Two mutations of this VOC, p.N501Y and p.A570D, are located within the RBD region of the S1 subunit. The p.N501Y mutation is probably the most important as it enhances the viral binding to the human ACE2 receptor, contributing to the transmissibility of this VOC [23–25].

**Table 1.** Epidemiological and genetic characteristics of the four SARS-CoV-2 variants of concern

WHO nomenclature	PANGO lineage	Emergence		Spike mutations		Impact	Efficacy of vaccines
		Country	Time	S1 subunit (1–685aa)	S2 subunit (686–1273aa)		
Alpha	B.1.1.7	UK	September 2020	p.H69del, p.V70del, p.Y144del, p.N501Y, p.A570D, p.D614G, p.P681H	p.T716I, p.S982A, p.D1118H	↑Transmissibility [26, 27] Affects more [31] ↓resistant to monoclonal Abs [32]	90% for BNT162b2 [79] ↓Neutralizing activity with mRNA-1273 [84] 72% for Ad26.COV2.S [87] 74% for ChAdOx1 nCoV-19 [82] 89.3% for NVX-CoV2373 [93]
Beta	B.1.351	South Africa	May 2020	p.D80A, p.D215G, p.L241del, p.L242del, p.A243del, p.K417N, p.E484K, p.N501Y, p.D614G	p.A701V	↑Resistant to Abs than Alpha VOC [35, 36] ↑The affinity with the ACE2 receptor [37]	75% for BNT162b2 [79] ↓↓↓Neutralizing activity with mRNA-1273 [83] 64% for Ad26.COV2.S [87] 81.5% for ChAdOx1 nCoV-19 [90] 49.4% for NVX-CoV2373 [93] ↓↓↓Neutralizing activity with Gam-COVID-Vac [96]
Gamma	P.1	Brazil	November 2020	p.L18F, p.T20N, p.P26S, p.D138Y, p.R190S, p.K417T/N, p.E484K, p.N501Y, p.D614G, p.H655Y	p.T1027I, p.V1176F	↑The affinity with the ACE2 receptor [40] ↑Transmissible [40] Resistant to neutralizing Abs [41, 42]	↓↓Neutralizing activity with mRNA-1273 [84] 50.4% for CoronaVac for symptomatic and 78% for mild SARS-CoV-2 infection [102]
Delta and Delta Plus	B.1.617.2	India	October 2020	p.T19R, (p.V70F)*, p.G142D, p.E156del, p.F157del, p.R158G, (p.A222V)*, (p.W258L)*, (p.K417N)* p.L452R, p.T478K, p.D614G, p.P681R	p.D950N	↑Transmissible [49] ↑The affinity with the ACE2 receptor [45, 46] ↑Resistant to monoclonal Abs [47]	↓↓BNT162b2 compared to Alpha [81] 67% for ChAdOx1 nCoV-19 [82] ↓with Covaxin [104]

aa, amino acids; Abs, antibodies; *mutations of Delta Plus variant; neutralizing activity = ↓: 1–3-fold reduction; ↓↓: 3–5-fold reduction; ↓↓↓: >5-fold reduction.

Their impact on natural infection and the efficacy of vaccines.

*In vivo* studies have shown that infected hamsters by p.N501Y and p.H69del, p.V70del lead to high viral levels in nasal secretions and upper airway, also confirmed in human airway epithelial cells [26]. The impact of p.P681H mutation is also the escape of the immunity [22]. Its location adjacent to the furin cleavage site, important for the membrane fusion of the virus, renders it significant. The p.P681H mutation is unique for Alpha VOC.

The Alpha VOC might be approximately 50% more transmissible than the wild-type SARS-CoV-2, probably due to the higher viral concentration than the previous circulating strains [27, 28]. It is calculated to elevate the infectious rate up to 7.5% per day [29]. Additionally, it seems to provoke higher mortality rate mainly in the community and not in hospitalised patients [30, 31]. Interestingly, the majority of the infected hospitalised patients were women, who have a higher rate of mortality [32].

A low resistance to monoclonal Abs against RBD or N-terminal of the spike protein of infected persons instead of vaccinated characterises the Alpha VOC [33].

### Beta VOC

In May of 2020, a second VOC of SARS-CoV-2 virus emerged in South Africa. This variant belongs to B.1.351 lineage, it is derived from the 20H (V2) clade and it was named as Beta VOC by WHO.

This VOC includes seven missense mutations and one three-amino acid deletion, the p.L241del, the p.L242del and the p.A243del (Table 1). The exact location of the deletion is unknown (241-243del or 242-244del), but the sequence of the Spike protein remains unchanged [34]. The p.N501Y and p.D614G mutations are common between Alpha and Beta VOCs. This VOC carried two new mutations within the RBD region of the Spike protein, the p.K417N and p.E484K.

A reinfection case of a 45-year-old woman has been described and supported by genomic evidence. This woman was infected the second time by the Beta VOC, which enhances the idea that this VOC evades the immune barrier [35]. Cele *et al*. also support that Beta VOC, probably due to the p.E484K mutation, might be more resistant to monoclonal Abs against SARS-CoV-2 proteins than Alpha VOC even in vaccinated or previously infected people [36, 37].

Nelson *et al*. have shown that the combination of p.E484K, p.K417N and p.N501Y, changing the structure of the spike protein, enhances the affinity of the virus with the ACE2 receptor, which probably renders this variant more contagious [38].

### Gamma VOC

In November of 2020, a third VOC of SARS-CoV-2 virus emerged in Manaus, the capital of Amazonas in Brazil [39, 40]. This variant belongs to P.1 lineage, it is derived from the 20J (V3) clade and it was named as Gamma VOC by WHO.

This VOC includes 12 missense mutations in the Spike protein (Table 1). Alpha, Beta and Gamma VOCs share the mutations p.N501Y in the RBD region and p.D614G near the furin cleavage site of the Spike protein. Beta and Gamma VOCs also share the mutations p.K417N and p.E484K in the RBD region of the spike protein.

Τhe triplet of p.K417T, p.E484K and p.N501Y is associated with increased virus binding to the human ACE2 receptor and transmissibility potential [41]. Antibodies produced by vaccines or natural infection are less likely to neutralise the P.1 VOC [42, 43]. Consequently, the emergence of the Gamma VOC raises concerns regarding the impact of the variant on the immunity and its possible invasion strategies.

### Delta VOC

In October of 2020, a forth VOC of SARS-CoV-2 virus emerged in India and until now it has spread to over 21 countries [44]. This variant belongs to B.1.617.2 lineage, it is derived from 21A clade and it was named as Delta VOC by WHO.

Delta differs a lot from the previous circulating variants as the only one common mutation is p.D614G. It carries two novel mutations, the p.L452R and p.T478K in the RBD region of Spike protein and a two-amino acid deletion within the S1 subunit, the p.E156del and p.F157del (Table 1).

*In vitro* studies have shown that the SARS-CoV-2 strains carrying the novel mutation p.G142D, located before the RBD region, could grow in the presence of a monoclonal antibody, thus this mutation may render the virus more resistant to the action of the immune system [45].

The p.L452R probably contributes to stronger binding of the virus to ACE2 receptor, enhancing the infectivity of this strain and to decreased recognition by monoclonal antibodies, probably escaping the immune response after vaccination [46–48]. Moreover computational analysis, regarding the infection dynamic of the SARS-CoV-2 variants, has shown that p.L452R mutation has a similar function as p.N501Y mutation [49]. This VOC is characterised by high levels of transmission [50].

The new lineage AY (sub-lineages: AY.1, AY.2, AY.4 and AY.4.2.), which derived from B.1.617.2 lineage and characterised by increased transmissibility, was named as ‘Delta Plus’. There are several additional spike mutations in Delta Plus variant such as p.V70F, p.A222V, p.W258L and p.K417N (Table 1) [51, 52]. As of October 2021, >2000 genome sequences of the Delta Plus variant have been identified in at least 46 countries, including the USA, India and the UK [53]. Compared to other VOC, it has been reported that Delta Plus resists monoclonal antibodies, such as Casirivimab and Imdevimab, as well as have increased affinity to the mucosa of lungs [54].

### Variants of interest

There are also known circulating variants that have a minor impact on the transmission, morbidity and mortality and they were named as VOI by WHO (Table 2).

**Table 2.** Epidemiological and genetic characteristics of the SARS-CoV-2 variants of interest

WHO nomenclature	PANGO Lineage	Emergence		Spike mutations		Impact	Efficacy of vaccines
		Country	Time	S1 subunit (1–685aa)	S2 subunit (686–1273aa)		
Epsilon	B.1.427/B.1.429	Southern California, USA	March 2020	p.S13I, p.W152C, p.L452R, p.D614G		↑Transmissibility [14] ↓Resistance to Abs from convalescent serum [14, 56]	↓↓Neutralizing activity with BNT162b2 and mRNA-1273 [42, 56] No differences in neutralizing activity with CoronaVac [109] 66% for Ad26.COV2.S [110] 55–81% for ChAdOx1 nCoV-19 [111, 112] 89% for NVX-CoV2373 [113]
Zeta	P.2	Brazil	April 2020	p.L18F, p.T20N, p.P26S, p.F157L, p.E484K, p.D614G	p.S929I, p.V1176F	No resistance to Abs from convalescent serum [114] ↑Resistance to bamlanivimab [115]	↓Neutralizing activity with BNT162b2 and CoronaVac [115, 116] 66% for Ad26.COV2.S [110] 55–81% for ChAdOx1 nCoV-19 [111, 112]
Eta	B.1.525	New York, USA	November 2020	p.Q52R, p.A67V, p.H69del, p.V70del, p.Y144del, p.E484K, p.D614G, p.Q677H	p.F888L	↑Resistance to Abs from convalescent serum [117, 118]	↓Neutralizing activity with BNT162b2 [117]
Theta	P.3	Philippines and Japan	February 2021	p.L241del, p.L242del, p.A243del, p.E484K, p.N501Y, p.D614G, p.P681H	p.E1092K, p.H1101Y, p.V1176F	↑Resistance to Abs similar to B.1.351 [119]	↓↓↓Neutralizing activity with BNT162b2 [119]
Iota	B.1.526	USA	November 2020	p.L5F, p.T95I, p.D253G, p.E484K, p.D614G	p.A701V	↓Resistance to Abs from convalescent serum and monoclonal Abs [14, 120]	↓↓Neutralizing activity with CoronaVac [109] 55–81% for ChAdOx1 nCoV-19 [111] 66% for Ad26.COV2.S [110] 94–95% for BNT162b2 and mRNA-1273 [112] 89% for NVX-CoV2373 [113]
Kappa	B.1.617.1	India	October 2020	p.E154K, p.L452R, p.E484Q, p.D614G, p.P681R	p.Q1071H	↓Susceptibility to Abs from COVID-19 patients serum than P.3 and B.1.351 [119]	↓Neutralizing activity with BNT162b2 [117] 78% for Covaxin [112] ↓Neutralizing activity with Covaxin [112]
Lambda	C.37	Peru	December 2020	p.G75V, p.T76I, p.R246del, p.S247del, p.Y248del, p.L249del, p.T250del, p.P251del, p.G252del, p.D253N, p.L452Q, p.F490S,p.D614G,	p.T859N	↑Transmissibility [121]	↓Neutralizing activity with BNT162b2 and mRNA-1273 [121] 55–81% for ChAdOx1 nCoV-19 [111] 66% for Ad26.COV2.S [112]
Mu	B.1.621	Colombia	January 2021	p.T95I, p.Y144S, p.Y145N, p.R346K, p.E484K, p.N501Y, p.D614G, p.P681H	p.D950N	↑Affinity with the ACE2 receptor [65] Unknown transmissibility [65]	↓Neutralizing activity with BNT162b2 [122]

aa, amino acids; Abs, antibodies; neutralizing activity = ↓: 1–3-fold reduction; ↓↓: 3–5-fold reduction; ↓↓↓: >5-fold reduction.

Their impact on natural infection and the efficacy of vaccines.

The Epsilon VOI, emerged in Southern California, belongs to B.1.427/B.1.429 lineage and 20/21C clade [55]. It has four mutations in spike protein, the commonest p.D614G, the Delta p.L452R and two novel ones, p.S13I and p.W152C in the NTD region of the spike protein. The p.S13I and p.W152C completely diminish the activity of monoclonal NTD-specific antibodies [56]. Although the two Delta mutations were associated with high virus levels and transmissibility, strong binding to human receptors and severe clinical manifestations [50, 57], they were not enough to categorise the Epsilon variant as VOC.

The Zeta VOI of P.2 lineage was first detected in Brazil in April 2020. It is named as VOI by WHO as it slightly negatively affects the NAc of antibodies [58]. This variant contains the following previously detected mutations in VOCs, p.L18F, p.T20N, p.P26S, p.E484K, p.D614G, p.V1176F and the new p.F157L and p.S929I.

The Eta VOI, detected in New York in November 2020, belongs to B.1.525 lineage and 21D clade. This variant contains the following previously detected mutations in VOCs, p.H69del, p.V70del, p.Y144del, p.E484K, p.D614G and the new mutations p.Q52R, p.A67V, p.Q677H and p.F888L in the spike protein [59].

The Theta VOI of P.3 lineage was detected in Philippines and Japan in February 2021. This VOI contains the previously detected mutations in VOCs, p.L241del, p.L242del, p.A243del, p.E484K, p.N501Y, p.D614G, p.P681H and the novel ones p.E1092K, p.H1101Y and p.V1176F [58].

The Iota VOI, emerged in the USA in November 2020, belongs to B.1.526 lineage and 21F clade. It carries six missense mutations in the spike protein, the most common p.D614G, the Beta mutations; p.E484K and p.A701V, and the novel mutations; p.L5F, p.T95I and p.D253G. Although it has spread rapidly within New York, it is not associated with severe disease and for this reason is categorised as VOI [60–62].

The Kappa VOI, closely related to Delta VOC, belongs to B.1.617.1 lineage and 21B clade. It carries six missense mutations in the spike, the common p.D614G, two Delta mutations; p.L452R and p.P681R, and the novel p.E154K, p.E484Q and p.Q1071H. This VOI carried a different amino acid substitution at the 484 position of the Spike protein from Glutamic acid to Glutamine than the p.E484K, which was in Beta and Gamma variants. Thus, the 484 Spike protein position may be susceptible to mutagenesis. However, Ferreira *et al*. found that the combination of p.L452R, p.E484Q and p.P681R mutations may have a lower efficiency in cell entrance [63].

The Lambda variant, detected in Peru and belonging to the 21G clade, was classified as VOI by WHO. It carries the previously detected mutation in all VOCs, p.D614G, and the novel ones p.G75V, p.T76I, p.D253N, p.L452Q, p.F490S, p.T859N and a seven-amino acid deletion; p.R246del, p.S247del, p.Y248del, p.L249del, p.T250del, p.P251del, p.G252del [64].

The recently emerged SARS-CoV-2 variant in Colombia of South America, the Mu variant belongs to 21H clade and according to WHO is a VOI. It carries nine mutations in the Spike protein, the common p.E484K, N501Y and p.D614G, the Iota mutation p.T95I, the Alpha variant p.P681H, the Delta variant p.D950N and the novel ones, p.Y144S (in the same amino acid position was previously reported a deletion in Alpha and Eta variants), p.Y145N and p.R346K [65].

### p.D614G: the most common mutation among the VOCs and VOIs

Early in the pandemic, Korber *et al*., who monitored amino acid changes in the SARS-CoV-2 spike protein included in a large sequence database, reported the emergence of a SARS-CoV-2 mutation that soon became the predominant circulating one worldwide, the p.D614G [66]. In May 2020, it was present in North America, Europe and Australia [66].

Recent *in vivo* and *in vitro* studies demonstrate that SARS-CoV-2 strains carrying the Glycine amino acid at 614 position of the spike protein are characterised by higher levels of infectivity and transmissibility in the respiratory tract, enhanced binding to ACE2 and increased replication in respiratory epithelial cells compared to the wild type amino acid (p.D614D) [67, 68]. British data from SARS-CoV-2 whole genome sequencing support that, even though substitution p.D614G is associated with infection in younger individuals and higher upper respiratory tract viral loads, there was no indication that patients infected with the p.D614G variant are associated with increased risk of clinical severity, hospitalisation or mortality rates [69].

Analysis of serologic reactivity of p.D614D and p.D614G showed the presence of cross-reactive IgG, IgM and IgA humoral immune responses in both variants of the spike [70]. There are no significant differences between the neutralisation degree of p.D614D and p.D614G, suggesting that serological assays will be able to detect both wild-type and mutated variants [71].

Based on the fact that the mutation p.D614G is outside of the RBD but near the furin cleavage site of Spike protein and the increase in RBD-mediated neutralisation, the mutation p.D614G is currently not expected to be a concerning barrier to vaccine development strategies [72]. However, the selective advantage of p.D614G highlights different transmission dynamics in population and the existence of unknown confounding factors require further monitoring [69].

Νow, this mutation seems to have established to viral genome as all SARS-CoV-2 VOCs and VOIs contain it.

## Effectiveness of SARS-CoV-2 vaccines against variants

Since COVID-19 was designated as a pandemic at the beginning of 2020, vaccines to prevent SARS-CoV-2 infection are considered the most promising approach for pandemic control. The development of vaccines has accelerated to an unprecedented pace, within only several months. Despite all the unpredicted changes in SARS-CoV-2 genome and the vaccines' variable efficacy and NAc rates against VOCs and VOIs, their main target, which is the prevention of severe disease, is retained for the time being [20].

### BNT162b2 and mRNA-1273

Two vaccines, BNT162b2 (Comirnaty; Pfizer, New York, USA and BioNTech, Germany) and mRNA-1273 (Spikevax; Moderna, United States National Institute of Allergy and Infectious Diseases and the Biomedical Advanced Research and Development Authority), based on the new mRNA technology were developed to prevent SARS-CoV-2 infection. Both vaccines have been found to be highly effective in clinical trials as well as in mass vaccination programmes with tolerable reactogenicity [73]. Recent studies have shown that there is an association of immunological response and NAc of BNT162b2 vaccine with several epidemiological and clinical characteristics [74, 75]. In addition, concerns have been raised regarding the impact of the emerging variants to the NAc and T-cell responses stimulated by the vaccine.

The produced NAbs with the BNT162b2 vaccine seems to be active against Alpha variant (B.1.1.7), but with a slight decrease in NAc compared to the wild-type strain first observed in Wuhan [76, 77]. Reduction in NAc against the B.1.1.7 variant may be attributed to its p.E484K spike substitution, since this mutation has previously been revealed to escape several monoclonal antibodies [77].

BNT162b2 vaccine also neutralises the virus carrying spike protein mutations found in B.1.351. A recent study showed that neutralizing titres in serum from vaccine recipients were three to four times lower for B.1.351 compared to those with wild-type virus, but still more enhanced than titres in convalescent individuals infected by wild-type virus [78].

In a study from Qatar that included more than 265 000 vaccinated individuals during the predominance of both B.1.1.7 and B.1.351, vaccine effectiveness (VE) of BNT162b2 was estimated at 90% for B.1.1.7 infection and 75% for B.1.351 infection [79]. However, a single dose of BNT162b2 was not associated with high effectiveness against both VOCs [79].

In a multicentre cohort study, in which over 23 000 healthcare workers in the UK participated, covering a time period when the B.1.1.7 variant was prevalent, VE against SARS-CoV-2 infection (both asymptomatic and symptomatic) was estimated at 70% 21 days or more following the first dose and 85% 7 days or more after the second dose of BNT162b2 vaccine, respectively [80]. Neutralizing response of BNT162b2 was three to fivefold lower against Delta variant (B.1.617.2) compared to Alpha variant (B.1.1.7) due to the activation of RBD and non-RBD humoral immunity evasion strategies [81]. In a case-control study, the effectiveness of two doses of BNT162b2 was 94% against B.1.1.7 and 88% against B.1.617.2 [82].

Similarly to BNT162b2, mRNA-1273 offers sufficient vaccine-elicited NAbs coverage against B.1.1.7 and includes up to 6–9-fold lower titre than with wild-type virus against B.1.351 [83] with an additional 2–3-fold times less antibody coverage against B.1.429 [83]. Recently, Wu *et al*. detected reductions of NAc by a factor of 1.2 in titres of NAbs against the B.1.1.7, 6.4 against the B.1.351 and 3.5 against the P.1 variant [84]. Compared to previous circulating strains, it seems that generation of NAbs levels is suggestively lower against B.1.351 and B.1.617.2 [85, 86]. Recently, McCallum *et al*. showed an approximately 2.5-fold decrease of NAc against B.1.427/B.1.429 in mRNA vaccine recipients' plasma, respectively [56].

### Ad26.COV2.S and ChAdOx1 nCoV-19/AZD1222

The Ad26.COV2.S (Janssen or Johnson & Johnson COVID-19 Vaccine; Janssen Vaccines in Leiden and Janssen Pharmaceuticals, Beerse, Belgium) and ChAdOx1 nCoV-19/AZD1222 (Covishield and Vaxzevria; Oxford University and AstraZeneca, Cambridge, United Kingdom) vaccines, both based on recombinant, replication-incompetent adenovirus vector technique, have raised controversial issues in the scientific community despite the high immunogenicity and efficacy rates, mainly regarding their association with the rare side effect of thrombotic thrombocytopenia and their neutralisation activity as well as its duration against most common SARS-CoV-2 VOCs [87–89].

In a phase III randomised control trial of the Ad26.COV2.S vaccine, VE was estimated at 66.9% in preventing moderate to severe SARS-CoV-2 infection. For instance, during the presence of p.D614G mutation in the USA, estimated VE rates were 74% and 72% 14 and 28 days after vaccination, respectively [87]. In South Africa, despite the high prevalence of the B.1.351 during the trial period, Ad26.COV2.S VE against moderate to severe disease was estimated at 52.0% 14 days and 64.0% 28 days after administration, respectively [87]. In Brazil, where the P.2 variant was prevalent, VE was estimated at 66% 14 days and 68% 28 days after administration, respectively [87]. These data are indicative of a significant heterogeneity in efficacy rates of Ad26.COV2.S between different regions worldwide.

Analysis of one of the randomised trials has shown considerable efficacy rates of ChAdOx1 nCoV-19 vaccine against variants that can evade human immune responses [90]. Despite the induction of low NAb responses against the B.1.1.7 *in vitro*, its efficacy in symptomatic COVID-19 patients infected by B.1.1.7 was not statistically different compared with other variants (70.4% *vs.* 81.5%) [90]. In South Africa, ChAdOx1 nCoV-19 did not reduce the rate of mild to moderate COVID-19 when the B.1.351 was predominant, with VE in symptomatic COVID-19 patients infected by the B.1.1.7 estimated at 70.4% and 81.5% in those infected by non-B.1.1.7 variants, respectively [90]. However, VE in asymptomatic COVID-19 patients infected by the B.1.1.7 was dropped to 28.9% [90].

Regarding the VE against SARS-CoV-2 VOCs in a study of 63 vaccinees, the detectability of NAc against approximately 60% of the study population due to immune evasion strategies in B.1.617.2 variant was confirmed [91]. In a case-control study, ChAdOx1 nCoV-19 VE was 74% against B.1.1.7 and 67% against B.1.617.2 [82].

### Other vaccines

NVX-CoV2373 (Novavax COVID-19 vaccine; Novavax, Gaithersburg, MD, USA and the Coalition for Epidemic Preparedness Innovations, Davos, Switzerland) is a recombinant protein nanoparticle vaccine composed of trimeric spike glycoproteins and a potent Matrix-M1 adjuvant [92]. In phase I/II randomised, placebo-controlled trial of healthy individuals <60 years old established that the vaccine, administrated in two doses 21 days apart, induced NAbs and CD4^+^ T-cell immune responses that exceed the magnitude of responses measured in convalescent serum [92]. In a phase III trial which enrolled more than 15 000 participants aged 18–84 years old in the UK and during the B.1.1.7 predominance, NVX-CoV2373 had an estimated efficacy of 89.3%, thus preventing from symptomatic SARS-CoV-2 infection at least 7 days following the second dose in seronegative individuals [93]. In South Africa, where most COVID-19 cases were infected by the B.1.351, it was shown that NVX-CoV2373 vaccine efficacy was 49.4% [94].

Gam-COVID-Vac (Sputnik V; Gamaleya Research Institute of Epidemiology and Microbiology, Moscow, Russia) was first developed in Russia and is based on two replication-incompetent adenovirus vectors (rAd26 and rAd5) that express a full-length spike glycoprotein [95]. An initial rAd26 dose is followed by a rAd5 boosting dose approximately 1.5–3 months later [95]. Phase III trials showed an estimated VE of 91.6% in preventing symptomatic SARS-CoV-2 infection after the administration of the second dose, inducing significant humoral and cellular immune responses in the study participants [95]. Compared to the wild-type virus, Gam-COVID-Vac was associated with a 6.8 and 2.8-fold reduction of NAc against B.1.351 and p.E484K, respectively [96].

AD5-nCOV (Convidecia or PakVac; CanSino Biologics, China) is based on a recombinant rAd5 vector that expresses the spike protein [97]. As a single dose, it elicits detectable immune responses approximately 28 days following the vaccination [97]. Even though a recent press release reported that CanSino vaccine was 75% effective in Pakistan, officially published clinical trials have not yet been implemented [98]. Since the availability of the vaccines is limited in China and other certain countries, including Mexico, Argentina, Chile, Russia and Pakistan, in parallel with the presence of multiple different VOCs [99, 100], more multicentre studies are required to determine neutralisation immune responses and VE to be better understood.

The CoronaVac (Sinovac COVID-19 vaccine; SinovacBiotech, Beijing, China), an aluminium hydroxide adjuvant COVID-19 vaccine developed in China, consists of an inactivated virus [101]. It is available in China, Brazil, Chile, Indonesia, Mexico and Turkey. Administrated in two doses 28 days apart, it elicits detectable immune responses with a good safety profile in adults aged 18–59 years [101]. Several studies performed in different countries report efficacy rates ranging from 50% to 91%. Interestingly, during the predominance of the P.1 variant in Brazil, there was only 50.4% reported efficacy rate at preventing symptomatic SARS-CoV-2 infection and 78% in mild cases [102]. In other studies, 91.25% efficacy rate at preventing symptomatic COVID-19 in Turkey and 65% in Indonesia were reported [100].

BBV152 (Covaxin; Bharat Biotech, India and Indian Council of Medical Research) is based on inactivated virus that was developed in India. It has an aluminium hydroxide and an imidazoquinoline molecule and a toll-like receptor agonist used to enhance cell-mediated responses [103]. It is administrated in two doses 29 days apart and is immunogenic in healthy individuals aged 18–55 years [103]. Compared to the wild-type virus, immunisation is accompanied by reduced NAb titres against the B.1.617 variant [104]. The presence of p.K417N in the Delta Plus variant is possible to increase antibody escaping strategies and it has been shown to have reduced neutralisation activity in BBV152 (Covaxin) vaccinated individuals [105].

WIV04 and HB02 (also known as BBIBP-CorV, Sinopharm COVID-19 vaccine; Sinopharm’s Beijing Institute of Biological Products, Beijing, China) are two inactivated, whole-virus vaccines using an aluminium hydroxide adjuvant that were based on two different SARS-CoV-2 isolates from patients in China [106, 107]. When administrated in two doses 28 days apart in individuals aged 18–80 years, they elicit significant neutralizing immune responses with good safety profiles [106, 107]. Phase III randomised clinical trials that included nearly 40 000 participants showed an estimated VE of 73% for WIV04 and 78% for HB02, starting 14 days post vaccination [108]. Despite those efficacy rates, further investigation regarding WIV04 and HB02 efficacy against VOCs is required.

## Conclusion

Although SARS-CoV-2 is an RNA virus which contains a viral protein with 3′-5′ exoribonuclease function, it has undergone many mutations since the beginning of the pandemic and has garnered worldwide attention due to its rapid and continuous spread. To date, four SARS-CoV-2 VOCs have been identified as they affect the transmission, clinical implications, morbidity and mortality. Although specific mutations of each VOC have been associated with some features, the same mutations in VOIs are not enough to classify them within VOCs, which pose a challenge to public health. A synergistic action of several different mutations may contribute to the clinical characteristics of infected patients, transmissibility or the escape from immunity. Currently available vaccines are capable of retaining NAc against VOCs, although the levels of NAbs vary. It still remains questionable whether VE against future SARS-CoV-2 variants will be preserved. The continuous surveillance of circulating SARS-CoV-2 variants will help in understanding the evolution of the virus and in developing more effective vaccines.

## Data Availability

All data presented in this review are collected from previously published papers and are available from the cited references.
